# Supplementary material for: SCAND1 Reverses Epithelial-to-Mesenchymal Transition (EMT) and Suppresses Prostate Cancer Growth and Migration
Source: Cells. 2022 Dec 10;11(24):3993. doi: 10.3390/cells11243993 (PMC9777339; doi:10.3390/cells11243993)
Supplement: Supplementary file 1 [file cells-11-03993-s001.zip › cells-2001504-supplementary.pdf]

Supplementary Materials

SCAND1 Reverses Epithelial-to-Mesenchymal Transition (EMT) and Suppresses Prostate Cancer Growth and Migration

Takanori Eguchi [1,*], Eva Csizmadia [2], Hotaka Kawai [3], Mona Sheta [1,4], Kunihiro Yoshida [1,5], Thomas L. Prince [6], Barbara Wegiel [2] and Stuart K. Calderwood [7,*]

[1] Department of Dental Pharmacology, Graduate School of Medicine, Dentistry and Pharmaceutical Sciences, Okayama University, Okayama 700-8525, Japan
[2] Division of Surgical Sciences, Department of Surgery, Cancer Research Institute, Beth Israel Deaconess Medical Center, Harvard Medical School, Boston, MA 02115, USA
[3] Department of Oral Pathology and Medicine, Graduate School of Medicine, Dentistry and Pharmaceutical Sciences, Okayama University, Okayama 700-85245, Japan
[4] Department of Cancer Biology, National Cancer Institute, Cairo University, Cairo 11796, Egypt
[5] Department of Oral and Craniofacial Surgery, Graduate School of Medicine, Dentistry and Pharmaceutical Sciences, Okayama University, Okayama, 700-8525, Japan
[6] Ranok Therapeutics, Waltham, MA 02451, USA
[7] Department of Radiation Oncology, Beth Israel Deaconess Medical Center, Harvard Medical School, Boston, MA 02115, USA

* Correspondence: eguchi@okayama-u.ac.jp (T.E.); scalderw@bidmc.harvard.edu (S.K.C.);
Tel.: +81 86 235 6661 (T.E.); +1 617 667 4240 (S.K.C.);
Fax: +81 86 235 6664 (T.E.), Fax: +1 617 667 4245 (S.K.C.)

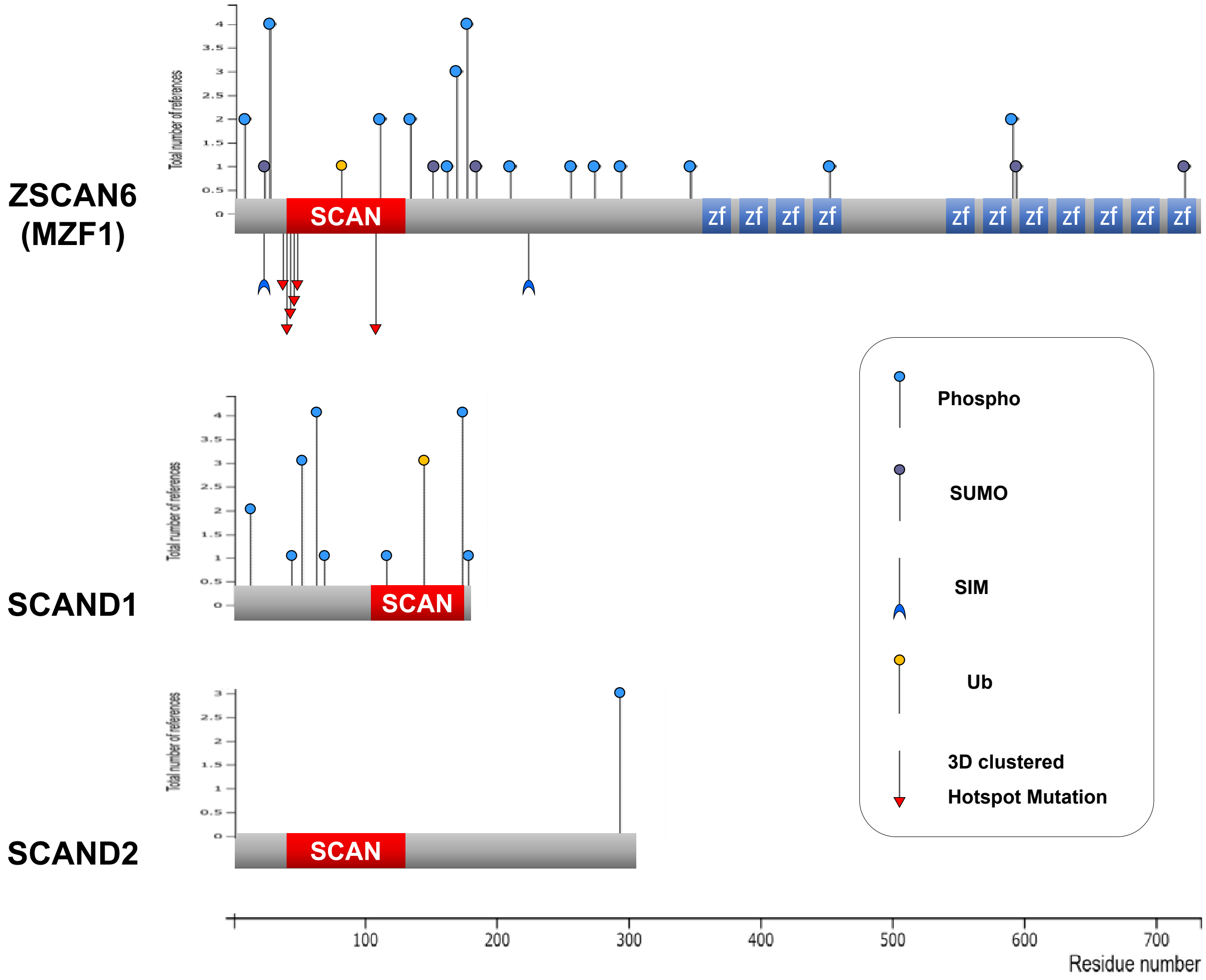

Figure S1. Post-translational modifications (PTM) and hotspot mutation in MZF1(ZSCAN6), SCAND1, and SCAND2. SCAN, SCAN domain. zf, zinc finger domain. Phospho, phosphorylation site. SUMO, sumoylation site. SIM, SUMO interaction motif. Ub, ubiquitination site. Some PTMs were shown in a ref. (Eguchi T, et al., 2015, J Cell Biochem).

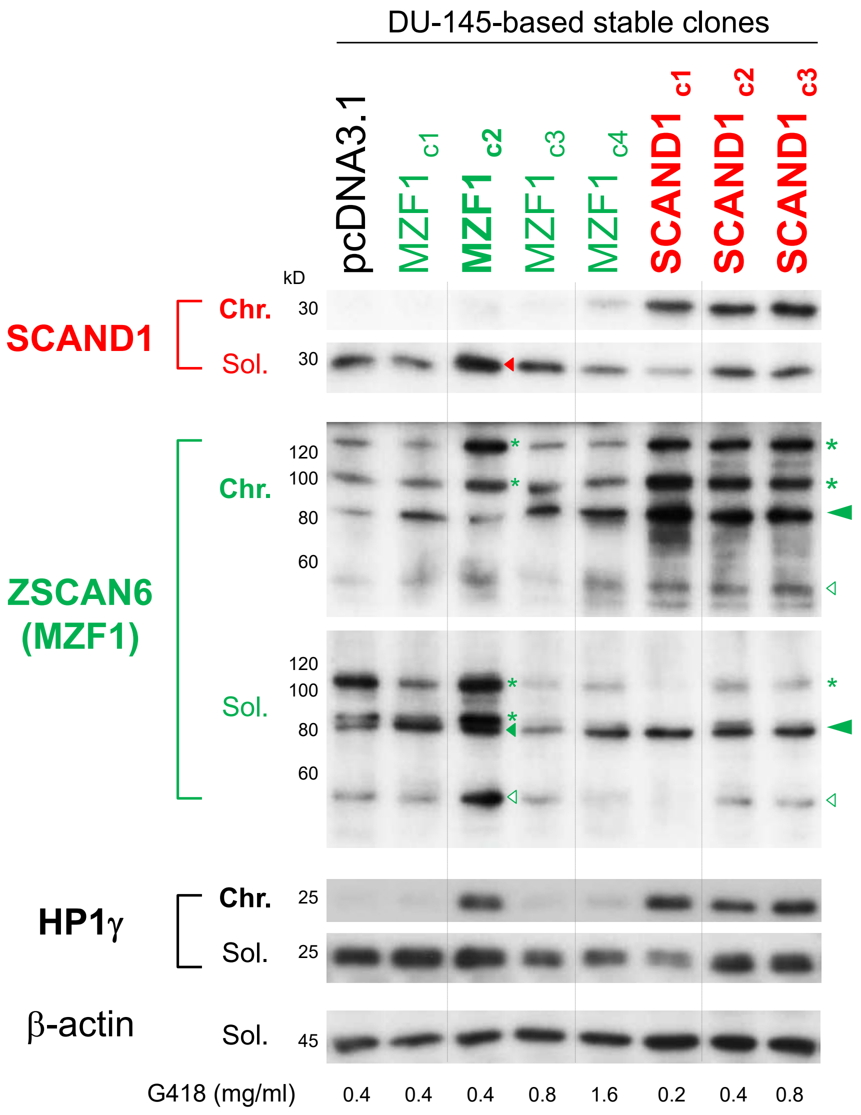

Figure S2. Western blotting of SCAND1, MZF1(ZSCAN6), and HP1γ in chromatin (Chr.) and soluble (Sol.) fractions in stable clones. G418 concentrations used for the selection were shown at the bottom. Green arrowheads indicate native full-length MZF1 (approx. 90 kD). Asterisks indicate potential MZF1 with PTMs, upshifted. White arrowheads indicate potential MZF1 isoform 3 or mutant (approx. 55 kD). See details of MZF1 PTMs and mutants in ref. (Eguchi T, et al., 2015, J Cell Biochem), PhosophoSitePlus, and NCBI protein database (ncbi.nlm.nih.gov/protein/XP_016882695.1).

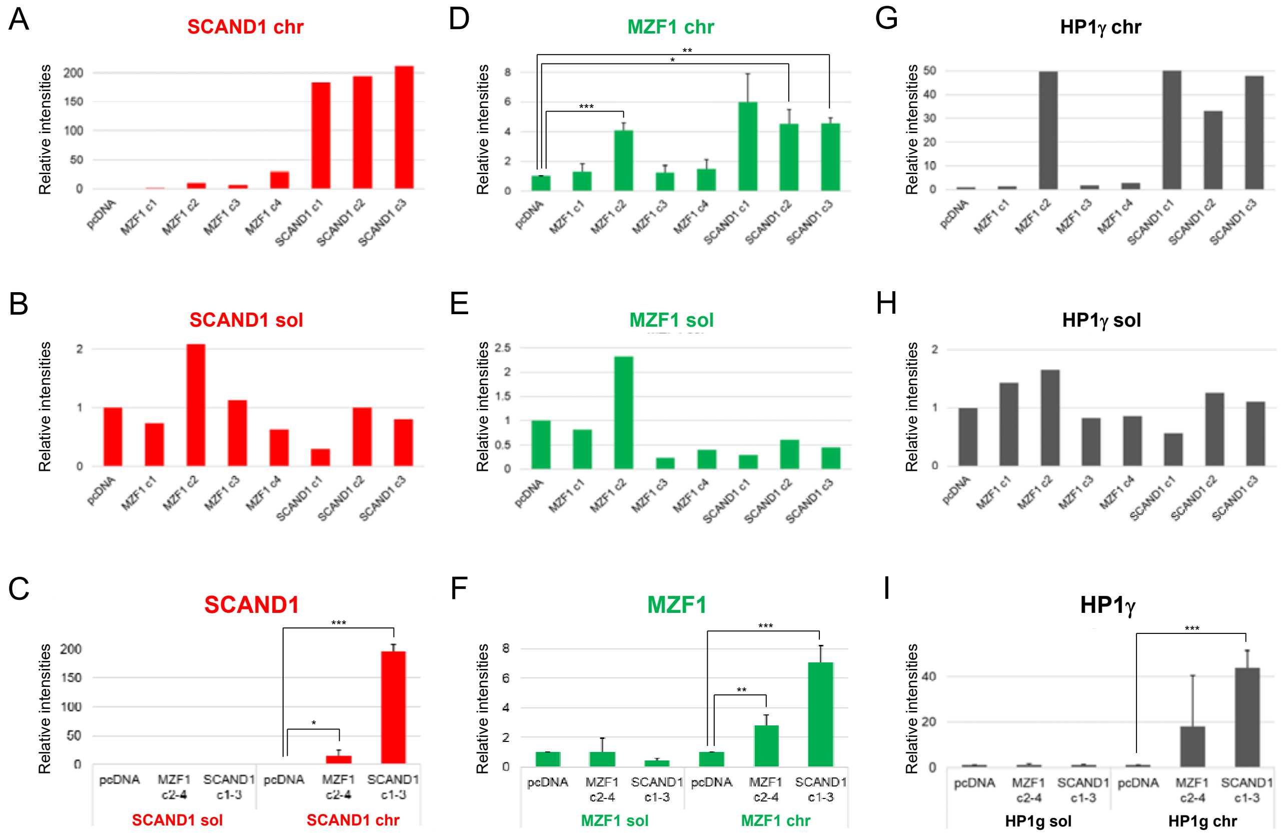

Figure S3. Expression levels of SCAND1 (A–C), MZF1(ZSCAN6) (D–F), and HP1γ (G–I) in stable clones. Relative intensities of bands in western blotting were quantified. sol, soluble fraction. chr, chromatin fraction. *$p<0.05$, n=3. **$p<0.01$, n=3. ***$p<0.005$, n=3.

Table S1. Correlated gene expression of SCAND1 and MZF1 vs. MAP3K, MAPK, CTNNB1, ZEB, and TGFBR.

**Correlated gene**	**SCAND1**			**MZF1**			Alternative name
	Spearman's correlation	p-Value	q-Value	Spearman's correlation	p-Value	q-Value	
**MAP3K2**	-0.715	3.37E-78	3.32E-76	-0.382	1.40E-18	1.36E-17	MEKK2
MAP3K1	-0.617	5.63E-53	1.15E-51	-0.347	2.13E-15	1.53E-14	MEKK1
MAPK1	-0.633	1.40E-56	3.43E-55	-0.548	5.07E-40	2.47E-38	ERK2
MAPK14	-0.564	8.68E-43	1.08E-41	-0.457	8.33E-27	1.70E-25	p38Alpha
CTNNB1	-0.621	5.48E-54	1.18E-52	-0.517	4.68E-35	1.67E-33	–
ZEB2	-0.433	5.85E-24	3.24E-23	-0.381	1.72E-18	1.66E-17	–
ZEB1	-0.309	2.35E-12	7.43E-12	-0.353	7.00E-16	5.28E-15	–
TGFBR2	-0.373	9.33E-18	3.88E-17	-0.362	1.14E-16	9.26E-16	–
TGFBR3	-0.334	2.65E-14	9.24E-14	-0.384	9.96E-19	9.85E-18	–
TGFBR1	-0.301	8.14E-12	2.50E-11	-0.487	1.09E-30	2.95E-29	–
